# 102 The Consequences of a Career in Burn Surgery: An Economic Analysis to Aid Prospective Clinicians

**DOI:** 10.1093/jbcr/irae036.101

**Published:** 2024-04-17

**Authors:** Jeffrey E Carter, Anastasiya Ivanko, Jonathan E Schoen, Herb A Phelan, William L Hickerson, Randy D Kearns

**Affiliations:** University Medical Center (LSU Health), New Orleans, LA; Louisiana State University Health Sciences Center- New Orleans, New Orleans, LA; University of Tennessee (Retired), Memphis, TN; University of New Orleans, New Orleans, LA; University Medical Center (LSU Health), New Orleans, LA; Louisiana State University Health Sciences Center- New Orleans, New Orleans, LA; University of Tennessee (Retired), Memphis, TN; University of New Orleans, New Orleans, LA; University Medical Center (LSU Health), New Orleans, LA; Louisiana State University Health Sciences Center- New Orleans, New Orleans, LA; University of Tennessee (Retired), Memphis, TN; University of New Orleans, New Orleans, LA; University Medical Center (LSU Health), New Orleans, LA; Louisiana State University Health Sciences Center- New Orleans, New Orleans, LA; University of Tennessee (Retired), Memphis, TN; University of New Orleans, New Orleans, LA; University Medical Center (LSU Health), New Orleans, LA; Louisiana State University Health Sciences Center- New Orleans, New Orleans, LA; University of Tennessee (Retired), Memphis, TN; University of New Orleans, New Orleans, LA; University Medical Center (LSU Health), New Orleans, LA; Louisiana State University Health Sciences Center- New Orleans, New Orleans, LA; University of Tennessee (Retired), Memphis, TN; University of New Orleans, New Orleans, LA

## Abstract

**Introduction:**

Many factors are assessed by prospective resident physicians and medical students when considering a career in healthcare. A rising awareness of educational costs, physician burnout, and well-being have many considering the economics of their decisions. Coupled with the projected physician shortage, burn surgeons are a vulnerable population consisting of less than 0.4% of all surgeons in the U.S. Our study examines the economic consequences of a career in burn surgery as compared to other surgical specialties.

**Methods:**

Using AAMC benchmark salary data, we calculated a net present value (NPV) for 12 surgical specialties. NPV was selected as it incorporates revenue and liabilities over the career period with the time value of money. Conservative assumptions from established references were used to determine resident salary, medical school debt, interest, opportunity costs, federal income taxes, and career duration (retirement at age 65). The model also assumes no delays in education or training, practice at an academic institution, and evaluated 2-year vs 1-year burn fellowship pathway against 11 other surgical careers: general, neurosurgery, orthopedic, pediatric, plastic and reconstructive, surgical oncology, cardiothoracic, transplant, trauma, urology, and vascular surgery.

**Results:**

Without loan forgiveness programs for medical school, physicians attending public institutions should expect to pay $509,173 over the life of the loan. Each year of fellowship after general surgery residency results in $257,641 of lost income after taxes. Neurosurgery, orthopedic, and cardiovascular performed >30% above the average NPV for all surgical specialties while surgical oncology and 2-year burn performed the worst at 28% and 23% below the average NPV, respectively. Selecting a 1-year burn fellowship improved performance to 11% below the average, while still outperforming trauma.

**Conclusions:**

The consequences of a career in healthcare are immense with few students or residents understanding the economic liabilities they assume because of historical perspectives that are unquestionably inaccurate in today’s climate. A career in burn surgery is rewarding but the economic considerations must be accounted for especially in a climate with limited resources. Our model takes many assumptions into consideration but does not take into account the productivity of burn surgeons relative to other surgical specialties making the inequity even more concerning. Additional research is needed to discern the complexity and intensity of burn care to aid future advocacy efforts.

**Applicability of Research to Practice:**

This study aids prospective burn docs to consider fellowship costs.

